# Alternative Splicing Profile and Sex-Preferential Gene Expression in the Female and Male Pacific Abalone *Haliotis discus hannai*

**DOI:** 10.3390/genes8030099

**Published:** 2017-03-08

**Authors:** Mi Ae Kim, Jae-Sung Rhee, Tae Ha Kim, Jung Sick Lee, Ah-Young Choi, Beom-Soon Choi, Ik-Young Choi, Young Chang Sohn

**Affiliations:** 1 Department of Marine Molecular Bioscience, Gangneung-Wonju National University, Gangneung 25457, Korea; kimmiaecho@gmail.com (M.A.K.); kio1231@naver.com (T.H.K.); 2 Department of Marine Science, College of Natural Sciences, Incheon National University, Incheon 22012, Korea; jsrhee@inu.ac.kr; 3 Department of Aqualife Medicine, Chonnam National University, Yeosu 59626, Korea; ljs@chonnam.ac.kr; 4 Phyzen Genomics Institute, Seongnam 13558, Korea; ahyoung@phyzen.com (A.-Y.C.); bschoi@phyzen.com (B.-S.C.); 5 Department of Agriculture and Life Industry, Kangwon National University, Chuncheon 24341, Korea

**Keywords:** abalone, *Haliotis discus hannai*, transcriptome, isoform, PIS system

## Abstract

In order to characterize the female or male transcriptome of the Pacific abalone and further increase genomic resources, we sequenced the mRNA of full-length complementary DNA (cDNA) libraries derived from pooled tissues of female and male *Haliotis discus hannai* by employing the Iso-Seq protocol of the PacBio RSII platform. We successfully assembled whole full-length cDNA sequences and constructed a transcriptome database that included isoform information. After clustering, a total of 15,110 and 12,145 genes that coded for proteins were identified in female and male abalones, respectively. A total of 13,057 putative orthologs were retained from each transcriptome in abalones. Overall Gene Ontology terms and Kyoto Encyclopedia of Genes and Genomes (KEGG) pathways analyzed in each database showed a similar composition between sexes. In addition, a total of 519 and 391 isoforms were genome-widely identified with at least two isoforms from female and male transcriptome databases. We found that the number of isoforms and their alternatively spliced patterns are variable and sex-dependent. This information represents the first significant contribution to sex-preferential genomic resources of the Pacific abalone. The availability of whole female and male transcriptome database and their isoform information will be useful to improve our understanding of molecular responses and also for the analysis of population dynamics in the Pacific abalone.

## 1. Introduction

The abalone (Gastropoda; Haliotidae) species is a marine gastropod that is herbivorous, single-shelled, and reef-dwelling. It is widely distributed throughout temperate and tropical coastal regions (~100 species) [1]. Of them, approximately 20 species are important for commercial aquaculture and wild fisheries worldwide as highly prized seafood items containing bioactive molecules (e.g., polysaccharides, proteins, fatty acids) that support health benefits beyond basic nutrition [2]. Global abalone production from farms reached 63,245 metric tons (MT) in 2010 after rapid development of aquaculture techniques [3]; total production increased to 85,344 MT in 2011 and 103,464 MT in 2013 [4]. Asian countries such as China, Korea, and Japan are currently major suppliers of abalone in the global market, while abalone culture is also growing in Europe (e.g., the UK, the Channel Islands, Ireland, France, and Spain) [4]. Particularly, Southeast Asia, along with China (80%) and Korea (12%), are the largest producers of farmed abalone in the world, whereas Japan and China are the major consumer regions [2,4].

Of the *Haliotis* species, the majority of Korean abalone production is composed of *Haliotis discus hannai*; its production is estimated to have increased over 60 times during the past 10 years and is predicted to reach over 10,000 MT by 2015 [4]. Because the abalone *H. discus hannai* is an organism of major economic interest, genomic resources and applications have recently focused on the understanding of the physiology, molecular adaptation, genetic selection, disease, defense mechanisms, and ecological genetic diversity of this species. Genes involved in numerous metabolic pathways such as innate immunity, cell stress and repair system, and antioxidant defense systems have been cloned and tested to determine their mRNA expression, translational inducibility, and molecular functions. Recently, the use of a genomics platform was successfully applied in *H. discus hannai*, although many research groups have continuously employed ‘-omics’ platforms (e.g., genomics, proteomics, linkage map development, Bacterial Artificial Chromosome (BAC) library construction) to understand the growth, development, reproduction, molecular adaptation, or innate immune response mechanisms upon environmental stressors or pathogen challenges in the *Haliotis* genus [5,6,7,8,9,10,11,12,13,14]. For *H. discus hannai*, a genetic linkage map was constructed as a potential application of marker-assisted selection in breeding programs using amplified fragment length polymorphism (AFLP) markers [15]. The genome size of *H. discus hannai* was measured as 1.84 pg of C value by using flow cytometry [16]. Recently, an RNA-sequencing application revealed various innate immune challenge-responsive transcriptomes in the *Vibrio parahaemolyticus*-infected *H. discus hannai* [17]. However, the public availability of the gene/genome information of *H. discus hannai* and the global response profiles of transcriptomes or proteomes are still scarce.

In this study, we analyzed the sex-preferential transcriptome of *H. discus hannai* and identified genes that are differentially expressed in female or male abalone. Specifically, we analyzed entire isoforms of female- or male-specific transcripts that are alternatively spliced at transcriptional control. To validate transcriptome profiles, quantitative real-time (RT)-PCR (qPCR) was employed using several sex-related genes in both female and male abalones. This transcriptome information will be useful as a first significant contribution to genomic resources for each sex of the Pacific abalone. Furthermore, it will provide valuable data for numerous forms of future research studies abalones.

## 2. Materials and Methods

### 2.1. Sample Preparation

Sexually mature male (131.3 g, 10.2 cm) and female (119.1 g, 9.5 cm) *H. discus hannai* were obtained from a public fish market on July 4th, 2015 at Wando-gun (Jeollanam-do, Korea), and identified to the subspecies using morphological characteristics [18,19]. The abalones were anesthetized with MgCl_2_ before dissection. To maximize the discovery of *H. discus hannai* gene pools, multiple tissues including ganglia, gills, intestine, hepatopancreas, muscle, and gonads were pooled, quickly frozen in liquid nitrogen, and stored at −80 °C until total RNA extraction. The sampling was performed in accordance with relevant institutional and national guidelines.

### 2.2. RNA Extraction and Library Construction

Pooled tissues of each sex were frozen in liquid nitrogen and homogenized with a glass pestle. Total RNA was extracted using the Hybrid-RTM kit (GeneAll Biotechnology Co., Seoul, Korea) according to the manufacturer's protocol. Total RNA was quantified by absorption of light at A260 and quality checked by analyzing the RNA Integrity Number (RIN) using a 2100 Bi system (Agilent Technologies Inc., Santa Clara, CA, USA).

Construction of full-length complementary DNA (cDNA) libraries and sequencing with the PacBio RSII platform (Pacific Bio-science Inc., Menlo Park, CA, USA) were performed at the National Instrumentation Center for Environmental Management (NICEM, Seoul National University, Seoul, Korea). Briefly, the first-strand cDNA was synthesized using the Clontech SMARTer PCR cDNA Synthesis kit (Takara Bio USA, Inc., Mountain View, CA, USA). The large-scale double-strand cDNA was generated with the optimal number of PCR cycles (for amplification) by the PCR cycle optimization test. The large-scale PCR products were purified and eluted to the optimal size (approximately 1–6 Kb) using the BluepippinTM System (Sage Science Inc., Beverly, MA, USA) and were re-purified using AMPure PB Beads (Pacific Biosciences Inc.) two additional times. A SMRTbell template library was prepared by repairing DNA damage, blunt end ligation, and annealing the SMRTbell adapter (according to the manufacturer’s protocol) using the SMRTbell library kit. The template was eluted to construct libraries of approximately 1–2 Kb, 2–3 Kb, and 3–6 Kb using the BluepippinTM system. Average molecular weight and concentration of the library were validated using an Agilent 2100 Bioanalyzer. All of the libraries were sequenced on the SMRTbell 1–2 Kb and 2–3 Kb libraries and the SMRTbell 3–6 Kb library using the PacBio RSII platform according to the manufacturer's instructions.

### 2.3. Gene Annotation and Isoform Analysis

The raw sequencing reads of full-length cDNA libraries were classified and clustered to transcript consensus using the ToFU (transcript isoforms: Full-length and Unassembled) pipeline (GitHub version, Pacific Biosciences of California, Inc. Menlo Park, CA, USA.) supported by PacificBiosciences. Briefly, the raw reads were classified to full-length (FL) and non-full-length (nFL) reads. The FL reads were clustered to isoform-level and were then used for re-clustering with nFL reads by Quiver, which is included in the ToFU pipeline. The initial transcript consensus sequences were filtered to obtain a high-quality isoform sequence with at least 99% accuracy (Figure S1). The final transcriptome isoform sequences were filtered by removing the redundant sequences with a CD-HIT that is a software for clustering and comparing protein or nucleotide sequence. Orthologous genes of the total transcript isoforms were analyzed by clustering the final transcript consensus contigs using a threshold of 0.99 identities with a cluster software (CD-HIT-est) of the CD-HIT-package v.4.6 (Weizhong Li's lab at UCSD, La Jolla, CA, US). In this software, the gene family, including paralogous and isoforms, is clustered into the orthologous groups. 

TransDecoder was used to identify candidate coding regions from the final transcriptome isoform sequence (The Broad Institute. Cambridge, MA, USA). The candidate coding regions were used for BLAST analysis against the UniProt and the NCBI non-redundant (nr) protein database (DB) to evaluate sequence similarity with genes of other species at an E-value cutoff of 1×10^-6^. All transcriptome information including isoform sequences of *H. discus hannai* is registered in the Phyzen DB (http://www.phyzen.com/haliotis/index.php).

### 2.4. Gene Ontology and Kyoto Encyclopedia of Genes and Genomes Pathway Analysis

Gene Ontology (GO; biological function, cell component, molecular function) and Kyoto Encyclopedia of Genes and Genomes (KEGG) pathway analysis of the contigs were performed using the GOstats program (Roswell Park Cancer Institute, Buffalo, NY, USA) and the Fisher’s Exact Test (*p* < 0.05), as implemented in the sequence annotation tool Blast2GO (BioBam Bioinformatics SL, Valencia, Spain). Three main categories for biological process, cellular component, and molecular function were obtained after comparing for similarities using default parameters. Gene annotation and GO analysis were performed at the NICEM, Seoul National University (Seoul, Korea). The assembled data were arranged based on read length, gene annotation, GenBank number, E-value, and species. In each section, the specific composition of GO terms was calculated and presented as a bar chart according to percentage.

### 2.5. Isoform Grouping

**Figure 1:**
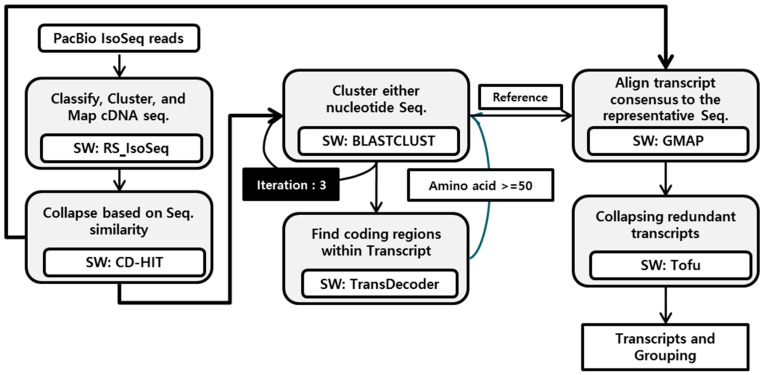
Schematic diagram of pipeline to isoforms of full-length complementary DNA (cDNA) sequence (PIS system).

In this study, we discovered the isoform groups using the pipeline to isoforms of full-length cDNA sequences (PIS) system that was developed with open software to assembly and blast using full-length cDNA sequence with no genome sequence DB. In the system, the total transcript isoform consensus sequences were aligned and mapped to the longest putative orthologous consensus to then search for the isoform level sequence using the ToFU pipeline and GMAP software (a genomic mapping and alignment program for mRNA and expressed sequence tags (EST)) [20] (Figure 1). Briefly, the total non-redundant high quality transcript isoform sequences were clustered using BLASTCLUST (NCBI, Bethesda, MD, USA). The longest reads were used as reference sequences to test for alternatively spliced isoform sequences; this was achieved by aligning them with an isoform-level consensus sequence. The reference sequences were re-updated to perform a three times replicate analysis and then re-clustered with an amino acid peptide sequence. The final alternative spliced isoform sequences were tested by alignment with transcript consensus sequences using GMAP and by filtering of redundant transcripts using ToFU pipelines.

### 2.6. Quantitative Real-Time RT-PCR

In order to test sex-preferential expression from each transcriptome, quantitative real-time RT-PCR (qPCR) was performed using six sex-preferential genes: *vitellogenin* (*VTG*), *forkhead box protein L2* (*FOXL2*), *condensin-2*, *sperm-associated antigen 6* (*SPAG6*), *protein fem-1 homolog C-like* (*FEM1-like*), and *tektin-1*, which are relatively well-characterized in marine invertebrates [9,21,22,23,24]. Based on the Minimum Information for Publication of Quantitative Real-Time PCR Experiments (MIQE) guidelines [25], transcriptional levels of each sex-preferential gene were validated using qPCR. Total RNA was extracted from the testes and ovaries of sexually mature abalone (four individuals from each sex) using the TriPure Isolation Reagent (Roche, Pleasanton, CA, USA). The maturity of gonads were examined by histological examination and revealed as being at the ripe and partial spent stages. After digestion of genomic DNA, 1 μg of total RNA was reverse transcribed using the PrimeScript RT reagent Kit (Takara Bio Inc., Shiga, Japan) with a gDNA eraser (Takara Bio Inc., Shiga, Japan). The resulting cDNAs were diluted, and an amount equivalent to 10 ng of starting RNA was assayed for mRNA expression analysis using *ribosomal protein 5* (*RPL5*) as the reference gene. SYBR-based qPCR reactions (SYPR Premix Ex Taq II, Takara, Japan) were performed on an Applied Biosystems 7500 Real-Time PCR System (Applied Biosystems, Foster City, CA, USA) using the following reaction conditions: 50 °C for 2 m, 95 °C for 10 m, followed by 40 cycles of 95 °C for 15 s, 60 °C for 1 m. The primer sets used in this study are listed in Table S1. A melting curve was generated at the end of the reaction to confirm an accurate amplification of the target amplicon. PCR efficiencies of the target and reference genes were verified. The relative mRNA expression was calculated according to the formula: 2^−(Ct target gene−Ct reference gene)^. All results are expressed as the mean ± S.E.M (standard error of mean).

### 2.7. Statistical Analysis

The SPSS software package (ver. 17.0; SPSS Inc., Chicago, IL, USA) was used for statistical analysis. Data are expressed as the mean ± S.E.M. Significant differences between female and male qPCR data were analyzed by the Student’s *t* test (two-tailed). *p* < 0.05 was considered to be significant.

## 3. Results and Discussion

### 3.1. Transcriptome Assembly and Gene Annotation

To establish a sex-specific transcriptome DB of the Pacific abalone *H. discus hannai*, we sequenced the mRNA of each female and male and abalone using pooled tissues (i.e., ganglia, gills, intestine, hepatopancreas, muscle, and gonads). After trimming and assembly, a total of 81 Mb nucleotide information (including 36,273 full-length cDNAs) of female and 60 Mb (including 29,275 full-length cDNAs) of male *H. discus hannai* were obtained using the PacBio RSII (Pacific Bio-science Inc.) sequencing platform with PIS system (Table S2). The lengths of female full-length cDNA ranged from 308 to 8377 bp with an average length of 2220 bp, whereas male full-length cDNAs showed an average length of 2059 bp and ranged from 308 to 8058 bp. The N50 values of those cDNAs were 2741 bp in female and 2477 bp in male. Subsequently, 18,692 and 15,271 high quality full-length consensuses were filtered from 22,494 (female) and 18,981 (male) full-length cDNAs, respectively, using CD-HIT platform in the absence of a redundant sequence. Finally, a series of bioinformatics, which comprised five platforms, defined 15,110 and 12,145 isoforms in female and male transcriptomes, respectively (Table 1; Figure S2).

Gene annotation of the whole transcripts was performed by BLASTx analysis using NCBI nr protein DB. The result showed that 15,110 female isoforms and 12,145 male isoforms had at least one positive BLAST hit (E-value < 1×10^-4^), representing 8274 and 6579 annotated genes, respectively (Tables S3 and S4). All data have been deposited in the Phyzen DB.

**Figure 2:**
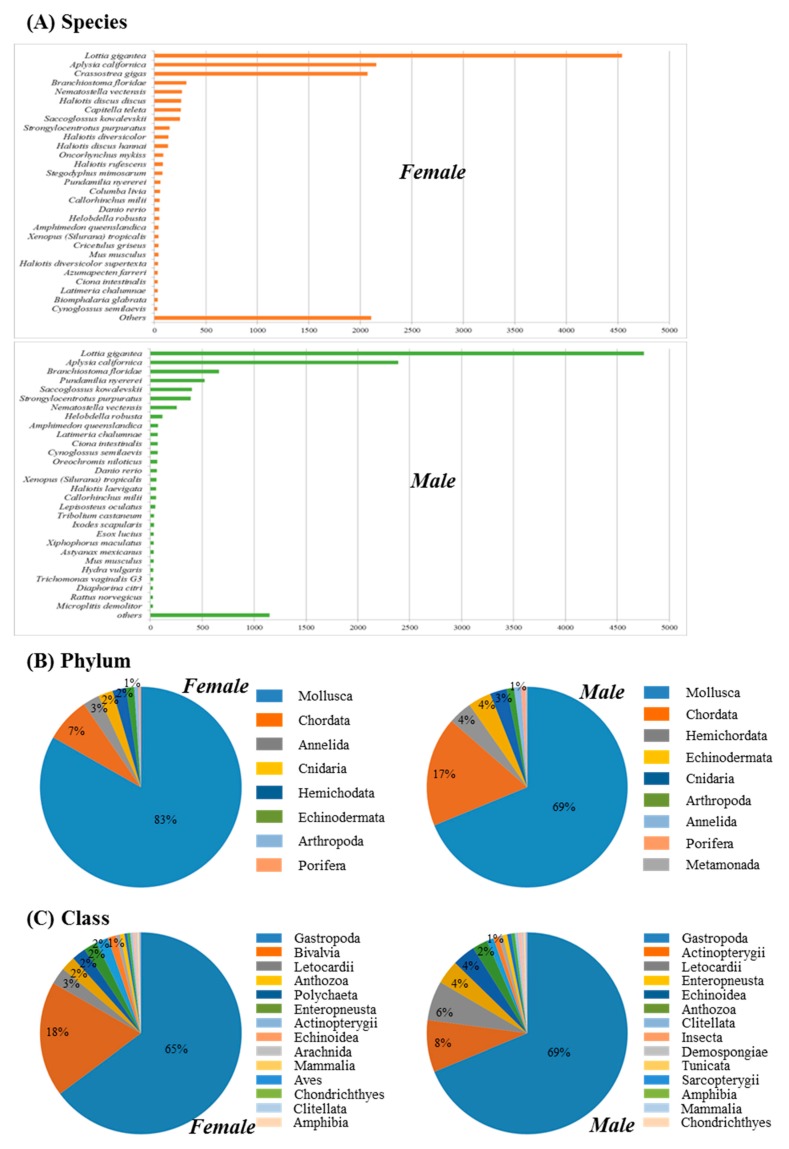
Homology searches of the female and male *Haliotis discus hannai* transcript contigs. (**A**) Number of BLAST hits; (**B**) top-hit phylum distribution; (**C**) top-hit class distribution.

Distribution analysis showed that 17 species had a BLAST hit with more than 50 transcripts in both sexes. Specifically, the owl limpet *Lottia gigantea* (Mollusca, Gastropoda) showed the highest similarity, with 4542 reads for the female abalone and 4759 reads for the male abalone (Figure 2A). Among the top hit species, 83% of female contigs (9478) and 69% of male contigs (7204) matched to the phylum Mollusca with a high similarity value (Figure 2B). In addition, 65% of female contigs (7375) and 69% of male contigs (7204) showed homology to Gastropoda at class level (Figure 2C). Thus, overall gene annotation results of *H. discus hannai* showed good agreement with their phylogenetic relationships.

**Figure 3:**
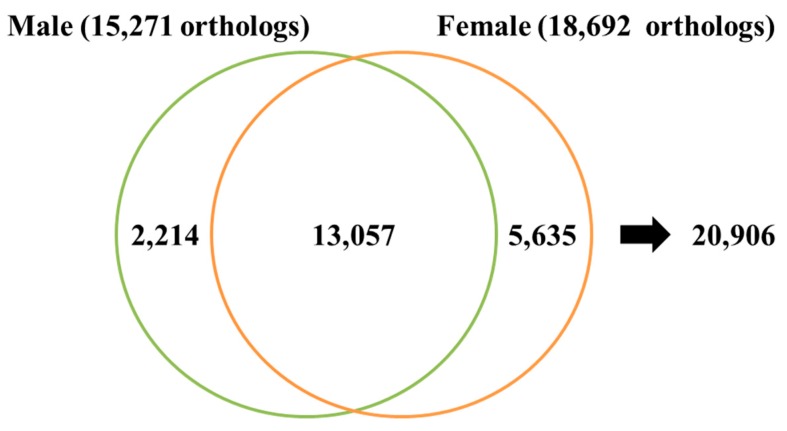
Gene discovery rate of each transcriptome database. Venn diagram to compare ortholog numbers annotated in the female and male *Haliotis discus hannai*.

Single copy orthologs were analyzed between female and male abalone using the reciprocal BLAST best-hit method (E-value < 1×10^-10^) with 18,692 female and 15,271 male nr high quality full-length cDNA sequences (Figure 3). Subsequently, 13,057 orthologs that could commonly serve in basic metabolism were determined between sexes of this species. We found that 85% female contigs had matches in the assembled contigs, whereas 91% matched with the male contigs (Table 2).

### 3.2. Functional Annotation

**Figure 4:**
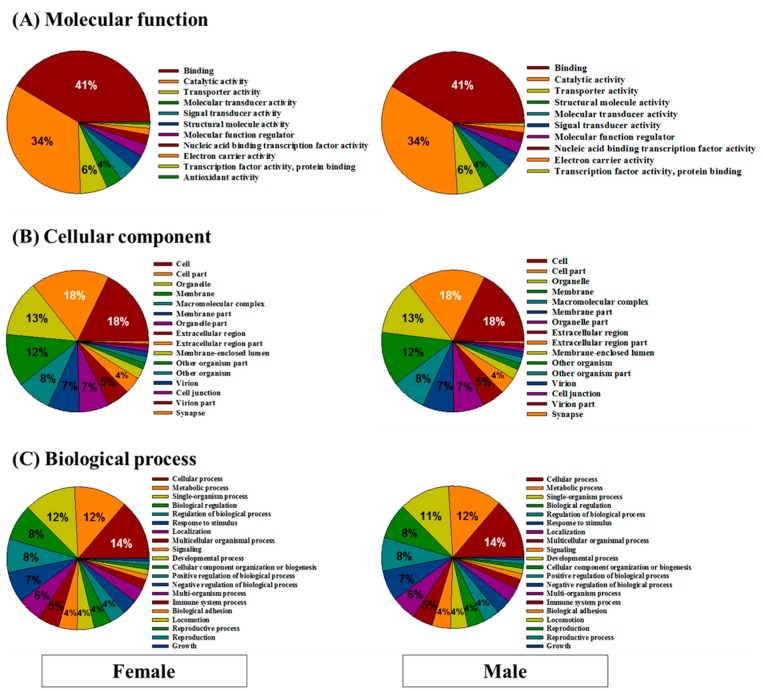
Gene Ontology (GO) analysis in terms of (**A**) molecular function, (**B**) cellular component, and (**C**) biological process that are enriched in the female and male *Haliotis discus hannai* transcript contigs.

To compare the transcriptome information of female and male *H. discus hannai*, a GO analysis in terms of cellular component, biological process, and molecular function was conducted using Blast2GO (Figure 4). Detailed GO distributions in three GO categories (i.e., biological process, cellular component, and molecular function) are incorporated in the Supplementary material (Tables S5–S10). The vast majority of genes were involved in binding (41% in both female and male) and catalytic activity functions (34% in both female and male) in the molecular function category (Figure 4A). In the cellular component class, most of the genes were related to cell (18% each), cell part (18% each), organelle (13% each), and membrane (12% each) in both female and male (Figure 4B). In the biological process class, most genes were categorized as being related to cellular processes (female: 14%; male: 14%), metabolic processes (female: 12%; male: 12%), and single-organism processes (female: 12%; male: 11%) (Figure 4C). Overall, very similar compositions (in percentage) were observed for both sexes in the three major categories using GO assignments. Although composition rates were relatively low in both sexes, there were some categories of biological processes that were differentially expressed between female and male abalones, including GO terms linked to the regulation of gene expression, positive regulation of transcription, reproduction, rhythmic process, and reproductive process. This finding indicates that some of the genes may be related to sex-specific roles.

To compare overall biological function between female and male transcripts, we classified all the assembled genes based on function using the KEGG pathway (Tables S11 and S12). KEGG analysis of the female and male *H. discus hannai* transcriptome revealed that the vast majority of KEGG pathways were involved in the ‘purine metabolism’ (female: #30; male: #30), followed by the ‘starch and sucrose metabolism’ (female: #25; male: #18), ‘pyruvate metabolism’ (female: #19) or ‘pyrimidine metabolism’ (male: #18), and ‘glycolysis/gluconeogenesis’ (female: #19; male: #17), suggesting that these metabolic pathways are actively expressed in both sexes. Similarly, the ‘purine metabolism’ was highly detected in the transcriptome DB of mixed organs from the South African abalone *H. midae* [26]. The ‘purine metabolism’ is important for providing basic components for nucleotides (i.e., DNA, RNA) and cellular energy sources like ATP. Most crucial members involved in sub-pathways (e.g., de novo biosynthesis of purine ring, salvage pathway) of the ‘purine metabolism’ are detected in both sexes (Figure S3). Thiamine is essential for carbohydrate metabolism and is also involved in thiazole and pyrimidine metabolism (i.e., B group of vitamin synthesis). Tissue contents of thiamin are closely associated with maximum growth of juvenile abalone [27]. The overall composition of the top ten female KEGG pathways was similar to that of its male counterpart. Thus, these results suggest the intactness of the *H. discus hannai* transcriptome, as the information dose not lack major functional GO categories or KEGG pathways compared to the transcriptomes of the genus *Haliotis*.

### 3.3. Isoform Analysis

Isoforms derived from alternative transcription start sites, alternative poly-adenylation, or alternative splicing were identified in *H. discus hannai* (Table 3). The numbers of genes covering at least two isoforms were as follows: 519 from 15,110 genes in female and 391 from 12,145 genes in male abalones (Tables S13 and S14). The numbers of isoforms were variable from 2 to 27 in female and from 2 to 52 in male abalones (Figure S2). 

**Figure 5:**
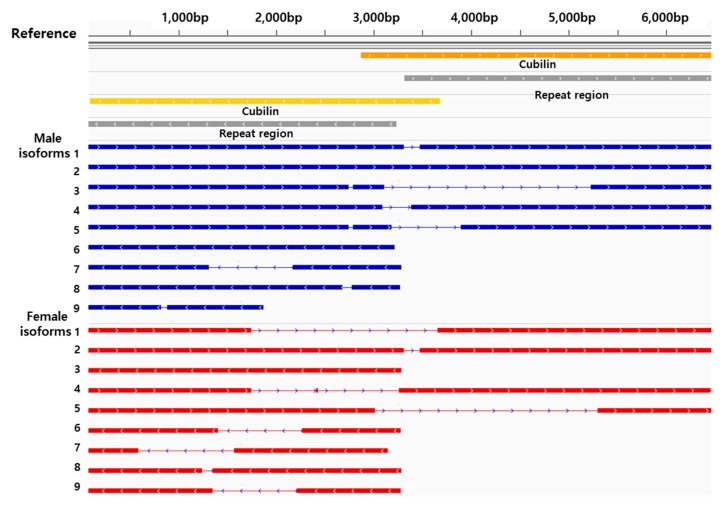
*Cubilin* isoforms in female and male Pacific abalone. *Cubilin* isoforms were determined by mapping consensus sequences to *cubilin* reference genes. Nine isoforms were defined in both female and male abalone, but only three isoforms showed the sequence in both sexes.

Of the identified isoforms in abalone, the cubilin protein has a high number of isoforms in both sexes (Table 4 and Table 5). In the GenBank DB, *cubilin* genes are annotated from 185 species; for example, there are five isoforms registered in humans. Thus, our discovery of isoforms using a PIS system could be useful for defining complex alternatively spliced patterns of certain genes. Surprisingly, the cubilin protein contains nine isoforms in both female and male abalone (Figure 5); from these, only three isoforms have the same sequence in both sexes. This is evidence that alternative splicing occurs differently in each sex. In addition, two putative *cubilin-like* genes, which are spliced from other loci by duplication, are observed to have nine and two isoforms in female and four and two isoforms in male abalones. Cubilin as an endocytic receptor is a large extracellular membrane protein (~450 kDa) [28]. In most cubilin proteins, two highly conserved domains, eight tandem epidermal growth factor domains followed by 27 tandem CUB (initially found in complement components C1r/C1s, Uegf, and bone morphogenic protein-1) domains harboring the intrinsic factor (IF)-cobalamin binding site (CUB domains 5–8), are included in extracellular modules [28,29]. Cubilin is associated with another large membrane-associated protein, megalin, to mediate the luminal uptake of a large number of proteins such as albumin. This protein complex plays a crucial role in the uptake of filtered carrier proteins such as vitamin D binding protein, retinol binding protein, transcobalamin, and transferrin [30]. Regarding the generally identifed functions of cubilin, we may suggest its putative diversified roles based on previous observations from mollusks including *Haliotis* sp.. Vitamin D metabolism is curcial in normal growth and shell mineralization of *H. discus hannai* [31,32]. Mollusks would have conserved and active retinol metabolism, as high retinyl ester storage capacity was observed with active retinoids in gastropod lineages [33,34]. Transcobalamin is a carrier protein which directly binds to cobalamin (vitamin B12). In fact, the cobalamin has been characterized as a nutrient source in most mollusks [35,36]. Tranferrin is a regulatory molecule of the innate immune system in mollusks that employs antimicrobial activity against a wide range of Gram-positive and -negative bacteria [37,38]. In *H. discus discus*, the in vivo antimicrobial property of transferrin through its Fe^3+^ binding ability was characterzied [39]. Although the functions of the cubilin protein in the renal proximal tubule of rodents and mammals are well established, information regarding the gene and its molecular mechanisms remains unclear in aquatic invertebrates. However, these results suggest that cubilin may be stronlgy involved with diverse metabolisms to maintain homeostasis and innate immunity in both female and male abalone. We assume that abalone cublin would have evolved with different transcription patterns by an alternative splicing mechanism as functional diversification. Our results also suggest that a series of bioinformatics platforms can be successfully applied to establish isoform information in the abalone.

### 3.4. Sex-Preferential Genes Expression

**Figure 6:**
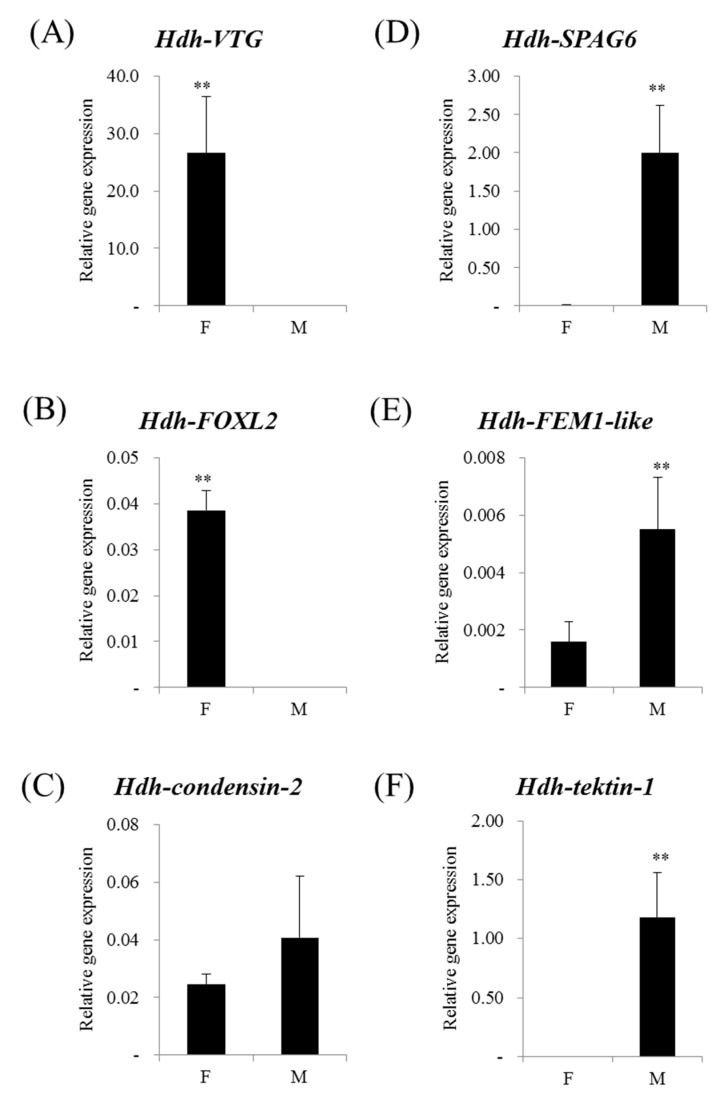
Relative mRNA expression profiles of the six selected genes from sexually mature *Haliotis discus hannai* by quantitative real-time RT-PCR analysis. The genes overexpressed in females were vitellogenin (*Hdh-VTG*) (**A**) and forkhead box protein L2 (*Hdh-FOXL2*) (**B**), but not condensin-2 (*Hdh-condensin-2*) (**C**). The genes overexpressed in males were sperm-associated antigen 6 (*Hdh-SPAG6*) (**D**), protein fem-1 homolog C-like (*Hdh-FEM1-like*) (**E**), and tektin-1 (*Hdh-tektin-1*) (**F**). Statistical changes were determined by the Student’s *t* test (two-tailed) and are denoted as follows: ** *p* < 0.01. M and F indicate female and male, respectively.

To validate our sex-specific isoform DB, we analyzed the mRNA expression profiles of six sex-preferential genes, which are suggested to be sex-specific markers in mollusks. Gender-related genes such as *VTG*, *FOXL2*, *condensin-2*, *SPAG6*, *FEM1-like*, and *tektin-1* were identified in the transcriptome DB, and their transcriptional levels in the same tissues (used in de novo assembly) were measured by the qPCR method. Of three female-related genes, *VTG* and *FOXL2* were predominantly detected in mature female tissues (Figure 6A,B), whereas *condensin-2* showed no significant difference between sexes (Figure 6C). In general, the yolk protein vitelline is synthesized from its precursor VTG and is accumulated in oocytes in most oviparous animals [40]. Although VTG is known to be synthesized in extraovarian tissues such as the fat body of insects, the hepatopancreas of decapod crustaceans, and the liver of vertebrates, many reports have suggested that the ovary can be a single source of VTG protein in mollusks [41,42,43,44,45,46,47,48]. The transcription factor *FOXL2* is preferentially expressed in the ovary and plays an important role in ovarian determination and development in vertebrates [49]. Ovary-preferential expression of *FOXL2* is also observed in several mollusks, as shown in the Pacific abalone [22,50,51,52,53]. Although a microarray-based analysis using the European clam *Ruditapes decussatus* showed that *condensin-2* is overexpressed in females during gonad development [21], we were not able to detect any significant differences in the Pacific abalone. 

The male-specific genes, *SPAG6*, *FEM1-like*, and *tektin-1* were highly expressed in mature male tissues (Figure 6D–F). SPAG6 is an axoneme central apparatus protein and has an essential role in the regulation of cilia/sperm flagella motility [54]. The *FEM1-like* gene is a component of the signal transduction pathway controlling sex determination in *Drosophila melanogaster* and *Caenorhabditis elegans* [55,56]. *Tektin-1*, a constitutive protein of microtubules in cilia, flagella, basal bodies, and centrioles is predominantly expressed in the testis and plays a role in spermatogenesis [57]. Male- or testis-preferential expressions of these genes has been consistently suggested in invertebrates [21,22,24,58]. Taken together, these studies strongly suggest the intactness of the sex-specific isoform DB of the Pacific abalone.

## 4. Conclusions

In this study, we characterized the transcriptome information for female and male *H. discus hannai*. We investigated differentially expressed isoforms involved in numerous signaling pathways and physiological metabolisms. Based on a comparative analysis of female and male abalone, the information obtained in this study represents the first significant contribution to sex-specific genomic resources, as well as isoform information. These data will provide an essential genomic reference that could be used for further diverse genetics- and physiology-based research using abalones.

## Figures and Tables

**Table 1:** Platforms for establishing gene sets of female and male *Haliotis discus hannai*.

Step	Data	Platform	Female	Male
1	High quality consensus sequence	RS_IsoSeq	22,494	18,981
2	Non-redundant representative sequence	CD-HIT	18,692	15,271
3	Reference isoforms	BLASTCLUST and TransDecoder	15,363	12,409
4	Final isoform transcriptome by combine representative sequence	GMAP and ToFU	15,792	12,718
5	Final gene set with representative isoforms	TransDecoder	15,110	12,145

**Table 2:** Ortholog statistics between female and male *Haliotis discus hannai*.

Number of Total Orthologs	13,057
Average identity (%)	99.33
Average coverage of female (%)	85.23
Average coverage of male (%)	90.63
Number of 100% coverage orthologs	502
Number of 100% identity orthologs	597

**Table 3:** Summary of the isoform information for *Haliotis discus hannai*.

Contig data	Female	Male
Total genes	15,110	12,145
Genes with no isoforms	14,591	11,754
Genes with at least two isoforms	519	391
Total length of genes with isoforms (bp)	1,599,611	1,166,159
Average length (bp)	3082	2982
Maximum length (bp)	8315	8058
Minimum length (bp)	741	847

**Table 4:** List of top-ranked genes containing over five isoforms in the female abalone.

Cluster ID	Length (bp)	#Isoform	Description	Matched Species	GenBank No.
F_Cluster00018	6565	27	deleted in malignant brain tumors one protein	*Columba livia*	ZDB-GENE-060228-6
F_Cluster11205	2162	11	-	-	-
F_Cluster00024	6380	9	PREDICTED: cubilin	*Haplochromis burtoni*	ZDB-GENE-060228-6
F_Cluster00089	4372	9	PREDICTED: cubilin-like	*Lingula anatina*	ZDB-GENE-060228-6
F_Cluster13261	1675	9	-	-	-
F_Cluster00812	3837	8	PREDICTED: cyclin-L1-like	*Crassostrea gigas*	H2U6Q2
F_Cluster00002	8315	7	PREDICTED: LOW QUALITY PROTEIN: sushi, von Willebrand factor type A, epidermal growth factor (EGF) and pentraxin domain-containing protein 1	*Callorhinchus milii*	F1MNH3
F_Cluster00829	3833	6	-	-	-
F_Cluster03162	3343	6	PREDICTED: serine/arginine-rich splicing factor 6-like isoform X1	*Crassostrea gigas*	A0A0D9SEM4
F_Cluster05356	3088	6	heterogeneous nuclear ribonucleoprotein L, partial	*Aplysia californica*	R4GHI6
F_Cluster11593	2114	6	-	-	-
F_Cluster00004	7437	5	hypothetical protein LOTGIDRAFT_214098	*Lottia gigantea*	NP_001116989.1
F_Cluster00011	6791	5	hypothetical protein AC249_AIPGENE2795	*Exaiptasia pallida*	F1NX90
F_Cluster10316	2268	5	PREDICTED: Na(+)/H(+) exchange regulatory cofactor NHE-RF1-like	*Biomphalaria glabrata*	XP_414851.3
F_Cluster12757	1916	5	-	-	-

**Table 5:** List of top-ranked genes containing over five isoforms in the male abalone.

Cluster ID	Length (bp)	#Isoform	Description	Matched species	GenBank No.
M_Cluster00016	6579	52	hypothetical protein cypCar_00021969, partial	*Cyprinus carpio*	ZDB-GENE-060228-6
M_Cluster00705	3598	11	PREDICTED: cyclin-L1-like	*Crassostrea gigas*	H2U6Q2
M_Cluster00017	6523	9	PREDICTED: cubilin	*Oreochromis niloticus*	ZDB-GENE-060228-6
M_Cluster01458	3359	8	PREDICTED: mesocentin-like	*Aplysia californica*	-
M_Cluster09253	1770	8	-	-	-
M_Cluster09585	1697	8	-	-	-
M_Cluster00226	3901	7	hypothetical protein LOTGIDRAFT_115468	*Lottia gigantea*	XP_002415964.1
M_Cluster00908	3513	7	serine-arginine protein 55	*Melipona quadrifasciata*	E1C270
M_Cluster00059	4569	5	hypothetical protein LOTGIDRAFT_200884	*Lottia gigantea*	NP_001040037.1
M_Cluster00931	3505	5	-	-	-
M_Cluster01425	3366	5	-	-	-
M_Cluster01740	3296	5	putative splicing factor, arginine/serine-rich 7	*Crassostrea gigas*	NP_064477.1
M_Cluster01748	3295	5	-	-	-
M_Cluster06621	2255	5	PREDICTED: tryptophan 2,3-dioxygenase-like	*Lingula anatina*	M3X838
M_Cluster09868	1649	5	-	-	-

## Supplementary Materials

The following are available online at www.mdpi.com/2073-4425/8/3/99/s1, Figure S1: Schematic chart of raw data processing and de novo assembly of the female and male *Haliotis discus hannai* transcriptome. To construct a high-resolution assembly, the SMART portal protocol was employed using an ICE algorithm and quiver algorithm, Figure S2: Gene distribution of the isoforms in female and male abalone. A total of 519 and 391 genes possess a minimum of two isoforms in female and male abalones, respectively, Figure S3: Example of the most highly matched pathway, ‘purine metabolism’, in (A) female and (B) male abalones using the KEGG pathway database, Table S1: Summary of the raw read sequencing information on *Haliotis discus hannai*, Table S2: BLASTx results of female abalone transcriptome, Table S3: BLASTx results of male abalone transcriptome, Table S4: GO term analysis of female abalone transcriptome: Molecular function, Table S5: GO term analysis of female abalone transcriptome: Cellular component, Table S6: GO term analysis of female abalone transcriptome: Biological process, Table S7: GO term analysis of male abalone transcriptome: Molecular function, Table S8: GO term analysis of male abalone transcriptome: Cellular component, Table S9: GO term analysis of male abalone transcriptome: Biological process, Table S10: KEGG analysis of female abalone transcriptome, Table S11: KEGG analysis of male abalone transcriptome, Table S12: List and description of genes including Isoforms from female abalone transcriptome, Table S13: List and description of genes including Isoforms from male abalone transcriptome, Table S14: Information on oligo primer sequences used in this study.

## References

[1] Estes J.A., Lindberg D.R., Wray C. (2005). Evolution of large body size in abalones (*Haliotis*): Patterns and implications. Paleobiology.

[2] Suleria H.A., Masci P.P., Gobe G.C., Osborne S.A. (2015). Therapeutic potential of abalone and status of bioactive molecules: A comprehensive review. Crit. Rev. Food Sci. Nutr..

[3] Cook P.A., Gordon H.R. (2010). World abalone supply, markets, and pricing. J. Shellfish Res..

[4] Cook P.A. (2014). The worldwide abalone industry. Mod. Econ..

[5] Sekino M., Hara M. (2007). Linkage maps for the Pacific abalone (genus *Haliotis*) based on microsatellite DNA markers. Genetics.

[6] Franchini P., van der Merwe M., Roodt-Wilding R. (2011). Transcriptome characterization of the South African abalone *Haliotis midae* using sequencing-by-synthesis. BMC Res. Notes.

[7] Huang Z.X., Chen Z.S., Ke C.H., Zhao J., You W.W., Zhang J., Dong W.T., Chen J. (2012). Pyrosequencing of *Haliotis diversicolor* transcriptomes: Insights into early developmental molluscan gene expression. PLoS ONE.

[8] Palmer M.R., McDowall M.H., Stewart L., Ouaddi A., MacCoss M.J., Swanson W.J. (2013). Mass spectrometry and next-generation sequencing reveal an abundant and rapidly evolving abalone sperm protein. Mol. Reprod. Dev..

[9] Mendoza-Porras O., Botwright N.A., McWilliam S.M., Cook M.T., Harris J.O., Wijffels G., Colgrave M.L. (2014). Exploiting genomic data to identify proteins involved in abalone reproduction. J. Proteom..

[10] Shiel B.P., Hall N.E., Cooke I.R., Robinson N.A., Strugnell J.M. (2015). De novo characterisation of the greenlip abalone transcriptome (*Haliotis laevigata*) with a focus on the heat shock protein 70 (HSP70) family. Mar. Biotechnol..

[11] Harney E., Dubief B., Boudry P., Basuyaux O., Schilhabel M.B., Huchette S., Paillard C., Nunes F.L. (2016). De novo assembly and annotation of the European abalone *Haliotis tuberculata* transcriptome. Mar. Genom..

[12] Jiang L., You W., Zhang X., Xu J., Jiang Y., Wang K., Zhao Z., Chen B., Zhao Y., Mahboob S. (2016). Construction of the BAC library of small abalone (*Haliotis diversicolor*) for gene screening and genome characterization. Mar. Biotechnol..

[13] Bathige S.D., Umasuthan N., Jayasinghe J.D., Godahewa G.I., Park H.C., Lee J. (2016). Three novel C1q domain containing proteins from the disk abalone *Haliotis discus discus*: Genomic organization and analysis of the transcriptional changes in response to bacterial pathogens. Fish Shellfish Immunol..

[14] Wang T., Nuurai P., McDougall C., York P.S., Bose U., Degnan B.M., Cummins S.F. (2016). Identification of a female spawn-associated Kazal-type inhibitor from the tropical abalone *Haliotis asinina*. J. Pept. Sci..

[15] Liu X., Liu X., Guo X., Gao Q., Zhao H., Zhang G. (2006). A preliminary genetic linkage map of the Pacific abalone *Haliotis discus hannai* Ino. Mar. Biotechnol..

[16] Adachi K., Okumura S.I. (2012). Determination of genome size of *Haliotis discus hannai* and *H. diversicolor aquatilis* (Haliotidae) and phylogenetic examination of this family. Fish. Sci..

[17] Nam B.H., Jung M., Subramaniyam S., Yoo S.I., Markkandan K., Moon J.Y., Kim Y.O., Kim D.G., An C.M., Shin Y. (2016). Transcriptome analysis revealed changes of multiple genes involved in *Haliotis discus hannai* innate immunity during *Vibrio parahemolyticus* infection. PLoS ONE.

[18] Ino T. (1952). Biological studies on the propagation of Japanese abalone (Genus *Haliotis*). Bulletin Tokai Reg. Fish. Res. Lab..

[19] Won S.-H., Kim S.-K., Kim S.-C., Yang B.-K., Lim B.-S., Lee J.-H., Lim H.K., Lee J.-S., Lee J.-S. (2014). The morphological characteristics of four Korean abalone species in *Nordotis*. Korean J. Malacol..

[20] Wu T.D., Watanabe C.K. (2005). GMAP: A genomic mapping and alignment program for mRNA and EST sequences. Bioinformatics.

[21] De Sousa J.T., Milan M., Bargelloni L., Pauletto M., Matias D., Joaquim S., Matias A.M., Quillien V., Leitão A., Huvet A. (2014). A microarray-based analysis of gametogenesis in two Portuguese populations of the European clam *Ruditapes decussatus*. PLoS ONE.

[22] Teaniniuraitemoana V., Huvet A., Levy P., Klopp C., Lhuillier E., Gaertner-Mazouni N., Gueguen Y., Le Moullac G. (2014). Gonad transcriptome analysis of pearl oyster *Pinctada margaritifera*: Identification of potential sex differentiation and sex determining genes. BMC Genom..

[23] Teaniniuraitemoana V., Huvet A., Levy P., Gaertner-Mazouni N., Gueguen Y., Le Moullac G. (2015). Molecular signatures discriminating the male and the female sexual pathways in the pearl oyster *Pinctada margaritifera*. PLoS ONE.

[24] Liu Y., Hui M., Cui Z., Luo D., Song C., Li Y., Liu L. (2015). Comparative transcriptome analysis reveals sex-biased gene expression in juvenile Chinese mitten crab *Eriocheir sinensis*. PLoS ONE.

[25] Bustin S.A., Benes V., Garson J.A., Hellemans J., Huggett J., Kubista M., Mueller R., Nolan T., Pfaffl M.W., Shipley G.L. (2009). The MIQE guidelines: Minimum information for publication of quantitative real-time PCR experiments. Clin. Chem..

[26] Picone B., Rhode C., Roodt-Wilding R. (2015). Transcriptome profiles of wild and cultured South African abalone, *Haliotis midae*. Mar. Genom..

[27] Zhu W., Mai K., Wu G. (2002). Thiamin requirement of juvenile abalone, *Haliotis discus hannai* Ino. Aquaculture.

[28] Fyfe J.C., Madsen M., Højrup P., Christensen E.I., Tanner S.M., de la Chapelle A., He Q., Moestrup S.K. (2004). The functional cobalamin (vitamin B12)-intrinsic factor receptor is a novel complex of cubilin and amnionless. Blood.

[29] Moestrup S.K., Kozyraki R., Kristiansen M., Kaysen J.H., Rasmussen H.H., Brault D., Pontillon F., Goda F.O., Christensen E.I., Hammond T.G. (1998). The intrinsic factor-vitamin B12 receptor and target of teratogenic antibodies is a megalin-binding peripheral membrane protein with homology to developmental proteins. J. Biol. Chem..

[30] Verroust P.J., Birn H., Nielsen R., Kozyraki R., Christensen E.I. (2002). The tandem endocytic receptors megalin and cubilin are important proteins in renal pathology. Kidney Int..

[31] Zhang W., Mai K., Xu W., Ai Q., Tan B., Liufu Z., Ma H. (2004). Effects of vitamin A and D on shell biomineralization of abalone *Haliotis discus hannai*, Ino. J. Shellfish Res..

[32] Zhang W., Mai K., Xu W., Tan B., Ai Q., Liufu Z., Ma H., Wang X. (2007). Interaction between vitamins A and D on growth and metabolic responses of abalone *Haliotis discus hannai*, Ino. J. Shellfish Res..

[33] Gesto M., Castro L.F., Reis-Henriques M.A., Santos M.M. (2012). Retinol metabolism in the mollusk *Osilinus lineatus* indicates an ancient origin for retinyl ester storage capacity. PLoS ONE.

[34] Gesto M., Ruivo R., Páscoa I., André A., Castro L.F., Santos M.M. (2016). Retinoid level dynamics during gonad recycling in the limpet *Patella vulgata*. Gen. Comp. Endocrinol..

[35] Watanabe F., Katsura H., Takenaka S., Enomoto T., Miyamoto E., Nakatsuka T., Nakano Y. (2001). Characterization of vitamin B12 compounds from edible shellfish, clam, oyster, and mussel. Int. J. Food Sci. Nutr..

[36] Tanioka Y., Takenaka S., Furusho T., Yabuta Y., Nakano Y., Watanabe F. (2014). Identification of vitamin B12 and pseudovitamin B12 from various edible shellfish using liquid chromatography-electrospray ionization/tandem mass spectrometry. Fish. Sci..

[37] Lambert L.A., Perri H., Halbrooks P.J., Mason A.B. (2005). Evolution of the transferrin family: Conservation of residues associated with iron and anion binding. Comp. Biochem. Physiol. B: Biochem. Mol. Biol..

[38] Liu J., Zhang S., Li L. (2009). A transferrin-like homolog in amphioxus *Branchiostoma belcheri*: Identification, expression and functional characterization. Mol. Immunol..

[39] Herath H.M., Elvitigala D.A., Godahewa G.I., Whang I., Lee J. (2015). Molecular insights into a molluscan transferrin homolog identified from disk abalone (*Haliotis discus discus*) evidencing its detectable role in host antibacterial defense. Dev. Comp. Immunol..

[40] Matozzo V., Gagné F., Marin M.G., Ricciardi F., Blaise C. (2008). Vitellogenin as a biomarker of exposure to estrogenic compounds in aquatic invertebrates: A review. Environ. Int..

[41] Osada M., Takamura T., Sato H., Mori K. (2003). Vitellogenin synthesis in the ovary of scallop, *Patinopecten yessoensis*: Control by estradiol-17β and the central nervous system. J. Exp. Zool. A Comp. Exp. Biol..

[42] Boutet I., Moraga D., Marinovic L., Obreque J., Chavez-Crooker P. (2008). Characterization of reproduction-specific genes in a marine bivalve mollusc: Influence of maturation stage and sex on mRNA expression. Gene.

[43] Matsumoto T., Yamano K., Kitamura M., Hara A. (2008). Ovarian follicle cells are the site of vitellogenin synthesis in the Pacific abalone *Haliotis discus hannai*. Comp. Biochem. Physiol. A: Mol. Integr. Physiol..

[44] Corporeau C., Vanderplancke G., Boulais M., Suquet M., Quéré C., Boudry P., Huvet A., Madec S. (2012). Proteomic identification of quality factors for oocytes in the Pacific oyster *Crassostrea gigas*. J. Proteom..

[45] Zheng H., Zhang Q., Liu H., Liu W., Sun Z., Li S., Zhang T. (2012). Cloning and expression of vitellogenin (*Vg*) gene and its correlations with total carotenoids content and total antioxidant capacity in noble scallop *Chlamys nobilis* (Bivalve: Pectinidae). Aquaculture.

[46] Ni J., Zeng Z., Kong D., Hou L., Huang H., Ke C. (2014). Vitellogenin of Fujian oyster, *Crassostrea angulata*: Synthesized in the ovary and controlled by estradiol-17β. Gen. Comp. Endocrinol..

[47] Wu B., Liu Z., Zhou L., Ji G., Yang A. (2015). Molecular cloning, expression, purification and characterization of vitellogenin in scallop *Patinopecten yessoensis* with special emphasis on its antibacterial activity. Dev. Comp. Immunol..

[48] Kim Y.-J., Lee N., Woo S., Ryu J.-C., Yum S. (2016). Transcriptomic change as evidence for cadmium-induced endocrine disruption in marine fish model of medaka, *Oryzias javanicus*. Mol. Cell. Toxicol..

[49] Georges A., Auguste A., Bessière L., Vanet A., Todeschini A.L., Veitia R.A. (2013). FOXL2: A central transcription factor of the ovary. J. Mol. Endocrinol..

[50] Naimi A., Martinez A.S., Specq M.L., Diss B., Mathieu M., Sourdaine P. (2009). Molecular cloning and gene expression of *Cg-Foxl2* during the development and the adult gametogenetic cycle in the oyster *Crassostrea gigas*. Comp. Biochem. Physiol. B: Biochem. Mol. Biol..

[51] Liu X.L., Zhang Z.F., Shao M.Y., Liu J.G., Muhammad F. (2012). Sexually dimorphic expression of foxl2 during gametogenesis in scallop *Chlamys farreri*, conserved with vertebrates. Dev. Genes Evol..

[52] Zhang N., Xu F., Guo X. (2014). Genomic analysis of the pacific oyster (*Crassostrea gigas*) reveals possible conservation of vertebrate sex determination in a mollusc. G3.

[53] Tong Y., Zhang Y., Huang J., Xiao S., Zhang Y., Li J., Chen J., Yu Z. (2015). Transcriptomics analysis of *Crassostrea hongkongensis* for the discovery of reproduction-related genes. PLoS ONE.

[54] Sapiro R., Kostetskii I., Olds-Clarke P., Gerton G.L., Radice G.L., Strauss J.F. (2002). Male infertility, impaired sperm motility, and hydrocephalus in mice deficient in sperm-associated antigen 6. Mol. Cell. Biol..

[55] Gaudet J., VanderElst I., Spence A.M. (1996). Post-transcriptional regulation of sex determination in *Caenorhabditis elegans*: Widespread expression of the sex-determining gene fem-1 in both sexes. Mol. Biol. Cell.

[56] Li W., Boswell R., Wood W.B. (2000). *mag-1*, a homolog of *Drosophila mago nashi*, regulates hermaphrodite germ-line sex determination in *Caenorhabditis elegans*. Dev. Biol..

[57] Tanaka H., Iguchi N., Toyama Y., Kitamura K., Takahashi T., Kaseda K., Maekawa M., Nishimune Y. (2004). Mice deficient in the axonemal protein Tektin-t exhibit male infertility and immotile-cilium syndrome due to impaired inner arm dynein function. Mol. Cell. Biol..

[58] Chen W., Liu Y.X., Jiang G.F. (2015). De novo assembly and characterization of the testis transcriptome and development of EST-SSR markers in the cockroach *Periplaneta americana*. Sci. Rep..

