# Supplementary material for: Alternative Splicing Profile and Sex-Preferential Gene Expression in the Female and Male Pacific Abalone Haliotis discus hannai
Source: Genes (Basel). 2017 Mar 8;8(3):99. doi: 10.3390/genes8030099 (PMC5368703; doi:10.3390/genes8030099)
Supplement: Supplementary file 1 [file genes-08-00099-s001.zip › Supplementary Figs-submitted.pptx]

## Slide 1
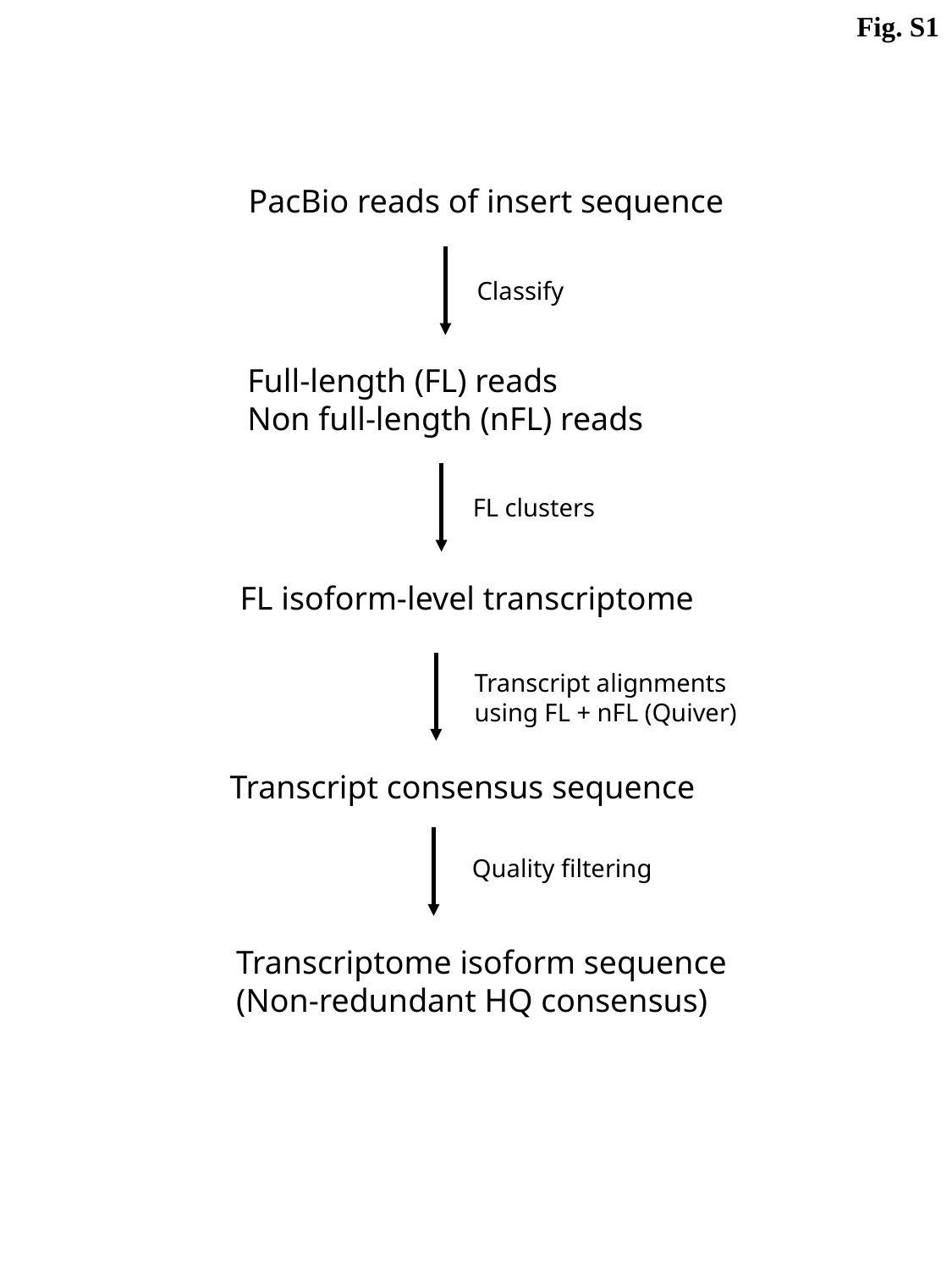

Fig. S1
PacBio reads of insert sequence
Classify
Full-length (FL) reads
Non full-length (nFL) reads
FL clusters
FL isoform-level transcriptome
Transcript alignments using FL + nFL (Quiver)
Transcript consensus sequence
Quality filtering
Transcriptome isoform sequence
(Non-redundant HQ consensus)

## Slide 2
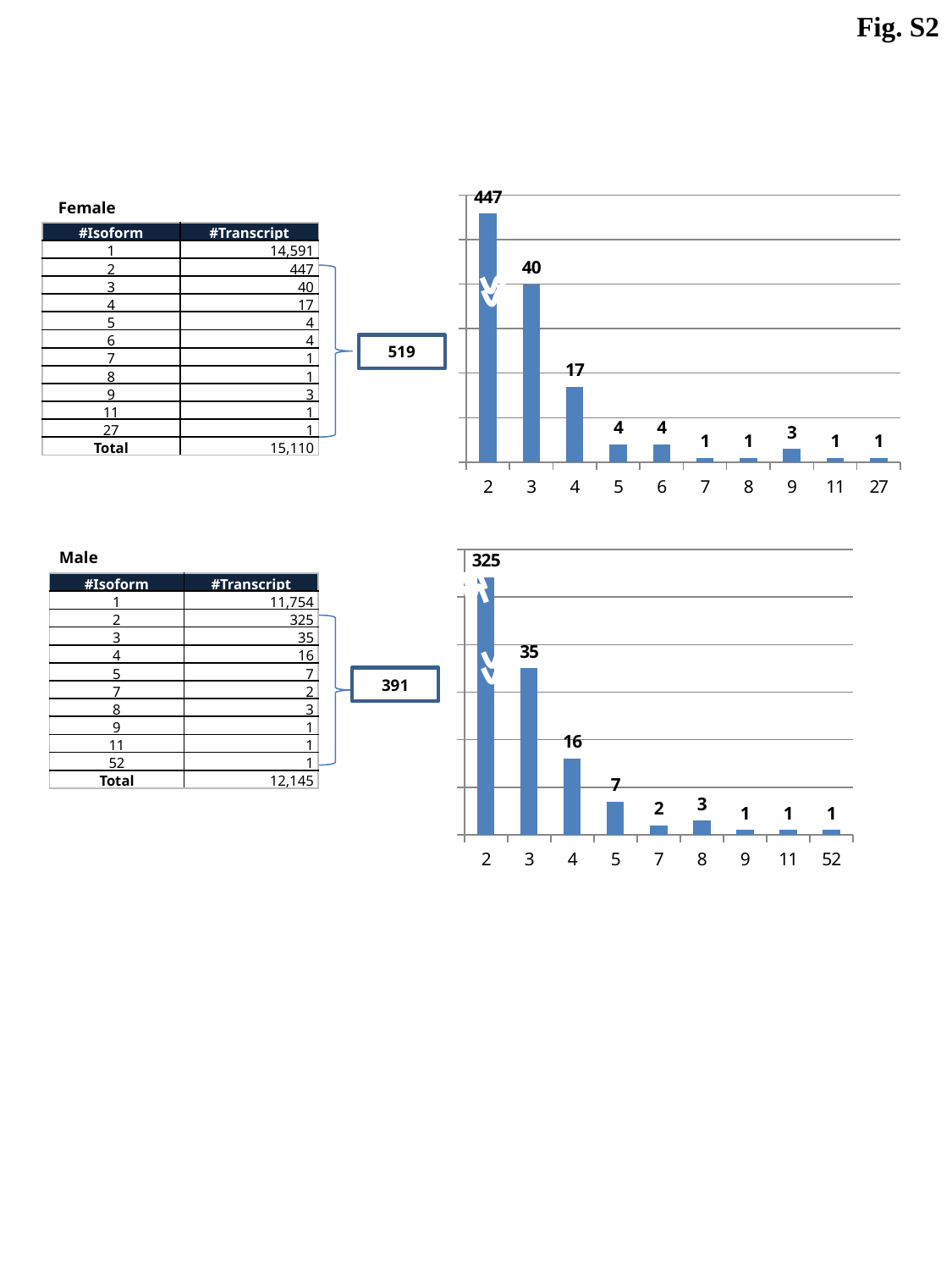

Fig. S2
### Chart
| Category | #Transcript |
|---|---|
| 2 | 55.875 |
| 3 | 40.0 |
| 4 | 17.0 |
| 5 | 4.0 |
| 6 | 4.0 |
| 7 | 1.0 |
| 8 | 1.0 |
| 9 | 3.0 |
| 11 | 1.0 |
| 27 | 1.0 |
Female
| #Isoform | #Transcript |
| --- | --- |
| 1 | 14,591 |
| 2 | 447 |
| 3 | 40 |
| 4 | 17 |
| 5 | 4 |
| 6 | 4 |
| 7 | 1 |
| 8 | 1 |
| 9 | 3 |
| 11 | 1 |
| 27 | 1 |
| Total | 15,110 |
519
Male
### Chart
| Category | #Transcript |
|---|---|
| 2 | 54.166666666666664 |
| 3 | 35.0 |
| 4 | 16.0 |
| 5 | 7.0 |
| 7 | 2.0 |
| 8 | 3.0 |
| 9 | 1.0 |
| 11 | 1.0 |
| 52 | 1.0 |
| #Isoform | #Transcript |
| --- | --- |
| 1 | 11,754 |
| 2 | 325 |
| 3 | 35 |
| 4 | 16 |
| 5 | 7 |
| 7 | 2 |
| 8 | 3 |
| 9 | 1 |
| 11 | 1 |
| 52 | 1 |
| Total | 12,145 |
391

## Slide 3
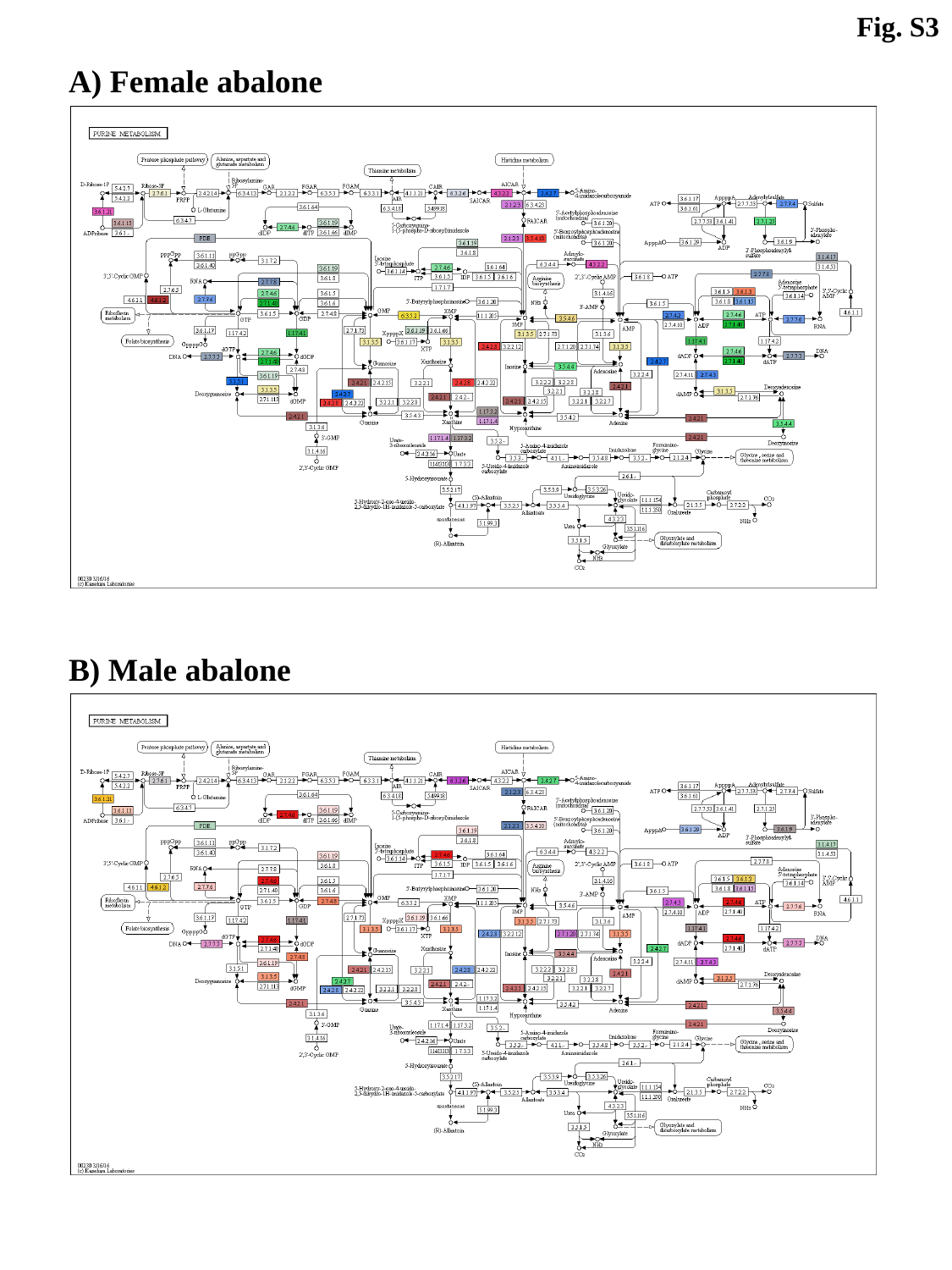

Fig. S3
A) Female abalone
B) Male abalone
